# Validity and Utility of Non-Invasive Prenatal Testing for Copy Number Variations and Microdeletions: A Systematic Review

**DOI:** 10.3390/jcm11123350

**Published:** 2022-06-10

**Authors:** Luca Zaninović, Marko Bašković, Davor Ježek, Ana Katušić Bojanac

**Affiliations:** 1 Scientific Centre of Excellence for Reproductive and Regenerative Medicine, School of Medicine, University of Zagreb, Šalata 3, 10 000 Zagreb, Croatia; zaninovicluca@gmail.com (L.Z.); davor.jezek@mef.hr (D.J.); ana.katusic@mef.hr (A.K.B.); 2 Children’s Hospital Zagreb, Ulica Vjekoslava Klaića 16, 10 000 Zagreb, Croatia; 3 Department of Histology and Embryology, School of Medicine, University of Zagreb, Šalata 3, 10 000 Zagreb, Croatia; 4 Department of Transfusion Medicine and Transplantation Biology, University Hospital Centre Zagreb, Kišpatićeva 12, 10 000 Zagreb, Croatia; 5 Department of Medical Biology, School of Medicine, University of Zagreb, Šalata 3, 10 000 Zagreb, Croatia

**Keywords:** non-invasive prenatal testing, microdeletion, copy number variation, cell-free DNA, validity, screening, prenatal diagnosis, molecular method

## Abstract

Valid data on prenatal cell-free DNA-based screening tests for copy number variations and microdeletions are still insufficient. We aimed to compare different methodological approaches concerning the achieved diagnostic accuracy measurements and positive predictive values. For this systematic review, we searched the Scopus and PubMed databases and backward citations for studies published between 2013 and 4 February 2022 and included articles reporting the analytical and clinical performance of cfDNA screening tests for CNVs and microdeletions. Of the 1810 articles identified, 32 met the criteria. The reported sensitivity of the applied tests ranged from 20% to 100%, the specificity from 81.62% to 100%, and the PPV from 3% to 100% for cases with diagnostic or clinical follow-up information. No confirmatory analysis was available in the majority of cases with negative screening results, and, therefore, the NPVs could not be determined. NIPT for CNVs and microdeletions should be used with caution and any developments regarding new technologies should undergo strict evaluation before their implementation into clinical practice. Indications for testing should be in correlation with the application guidelines issued by international organizations in the field of prenatal diagnostics.

## 1. Introduction

In response to the growing appreciation of the incidence of and a better understanding of the importance of submicroscopic copy number variations and cytogenetic abnormalities other than common aneuploidies in recent years, laboratories have begun developing the ability to identify smaller cytogenetic changes using cell-free DNA. Fetal DNA analysis is the only method for detecting these disorders. The correlation between an elevated risk for pathological copy number variations and increased nuchal translucency, as well as altered serum levels of PAPP-A and free β-HCG, was noticed [1,2]. Other than these, the main indications for wide NIPT analysis are previous children with chromosomal alterations, the sonographic detection of fetal abnormalities, and a history of family members testing positive for chromosomal or genetic disorders [3]. Currently, there are two non-invasive approaches; one targets a handful of clinically significant microdeletions, and the other sets a size cutoff threshold for genome-wide copy number variations that are often the cause of microdeletions. Peters et al. were the first to apply this methodology in prenatal screening for fetal CNVs in 2011 [1,2,4]. In contrast to the convincing evidence for cell-free DNA-based screening for trisomies 21, 18, and 13, valid data concerning the accuracy and positive predictive values of most of these additional tests are still missing. There is a concern that a low incidence and a potentially much lower PPV for CNVs and microdeletions, resulting in a high false-positive rate, will increase the number of unnecessary invasive tests performed, especially taking into consideration that, due to the lack of official guidelines, the test is often performed on a population of pregnant women who are not preselected [2]. There are many controversies regarding this topic since clinical validation for cell-free DNA microdeletion and CNV testing has been sacrificed in a commercial race to expand indications for noninvasive testing. Many argue that the reliability and accuracy of NIPT for the detection of such conditions have not been subjected to the rigor necessary to make this a valid clinical test [5]. Any recommendations for the use of these tests should be based on clinical research evidence. There are several reviews on this topic and one systematic review that included seven cohort studies, but we intend to offer a more comprehensive and up-to-date overview of the literature available on this subject [2,6,7,8,9].

The objective of this systematic review was to evaluate the accuracy and reliability of non-invasive prenatal testing for CNVs and microdeletions and to compare different approaches, taking into account molecular methods of sample analysis and the assumed risk for the studied population of pregnant women as well as the biological characteristics of the analyzed submicroscopic anomalies.

## 2. Materials and Methods

### 2.1. Study Design and Search Strategy

The study was performed according to Preferred Reporting Items for Systematic-Reviews and Meta-Analysis (PRISMA) statement.

**Figure 1:**
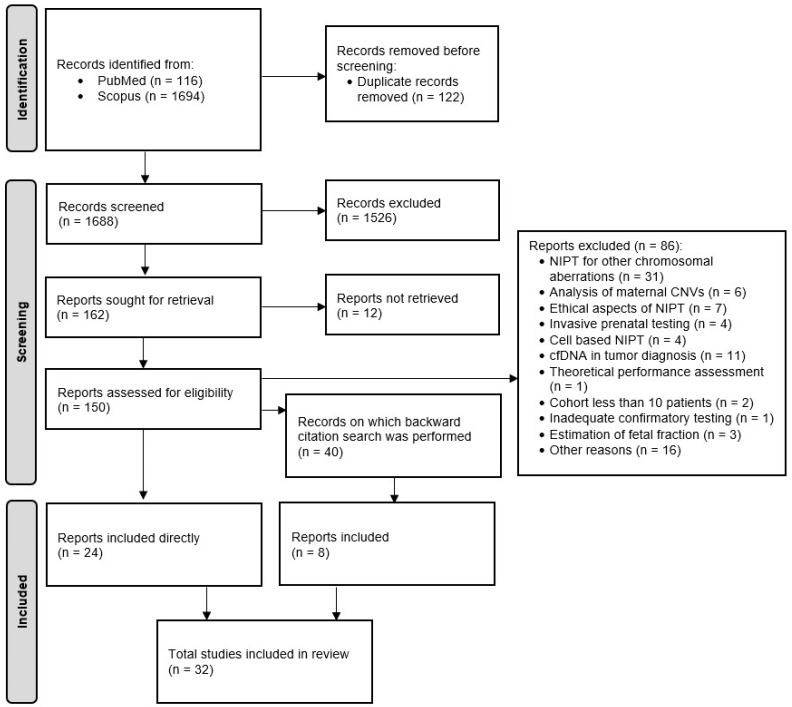
PRISMA flow diagram.

On 4 February 2022, we searched the Scopus and PubMed databases. The search combinations used included the Boolean operators “AND” and “OR” in combination with the following MeSH and free text terms: [(prenatal diagnoses) OR (prenatal diagnosis)] AND [(non-invas *) OR (noninvas *) OR (non invas *)] AND [(cell-free DNA) OR (cell free DNA) OR (cfDNA) OR (cffDNA)] AND [(test *) OR (valid *)] AND [(microdel *) OR (copy number varia *)]. Neither search filters nor text analysis tools were used. The study selection process is described in Figure 1.

### 2.2. Inclusion and Exclusion Criteria

We included a total of 32 study reports. To be included, a report had to contain information about the validity or utility of cfDNA-based non-invasive prenatal testing for fetal CNVs and microdeletions. Articles reporting solely on the application of NIPT for the detection of other chromosomal aberrations were excluded. Furthermore, reports in which the validity of the test was not confirmed by invasive testing or statistically expressed were excluded. Articles describing ethical aspects of NIPT as well as the ones addressing the usage of cfDNA-based methodology in the terms of oncology are valuable but not relevant to the topic and we, therefore, excluded them. We restricted our selection to English-language articles only and those published from 2013 onwards, as that is when this type of screening test first became clinically available.

### 2.3. Screening Process and Critical Appraisal

Studies were selected in a four-stage process. The first step was to assess their eligibility based on the title and abstract. Two researchers independently reviewed the titles and abstracts of half of the records each. In the second step, the same two researchers independently screened full-text articles for inclusion. In case of disagreement, the consensus was reached by discussion. Study reports were directly included in our systematic review, and a backward citation search solely for study reports was performed on the other articles (reviews, book chapters, case reports, commentaries, debate reports). As in the first step, two researchers independently reviewed titles and abstracts, then screened full-text reports. Subsequently, the data were collected by one independent researcher (see flow diagram summarizing the selection of studies for inclusion in the systematic review). The review protocol was registered with the International Prospective Register of Systematic Reviews (PROSPERO, ID334674).

## 3. Results

### 3.1. Search Results

Overall, 1810 references were collected in Mendeley. After the exclusion of duplicates, 1688 articles remained. Based on the titles and abstracts, 1526 were excluded. Of the remaining 162 articles, 24 study reports were directly included, and a backward citation search was performed on the 40 selected articles, resulting in eight study reports appointed for inclusion in the review.

### 3.2. Chromosomal Aberrations of Interest

A total of 32 studies were included in this systematic review (Table 1). Of those, 21 studies explored screening possibilities for microdeletion syndromes using cell-free DNA. The most frequently analyzed pathologies were the DiGeorge (22q11.2 del), Cri-du-chat (5p), Prader–Willi/Angelman (15q del), 1p36 deletion, and Wolf–Hirschhorn (4p del) syndromes [10,11,12,13,14,15,16,17,18,19,20,21,22,23,24,25]. Helgeson et al. additionally screened for the 8q and 11q (Jacobsen) deletion syndromes and Koumbaris also checked for Smith–Magenis (17p del) syndrome [15,19]. Two studies focused on the Southeast Asian (SEA) deletion (20 kb size) [26,27], which is the cause of α0-thalassemia and Bart’s hydrops fetalis. Three studies estimated possibilities for the genome-wide detection of microdeletions [28,29,30]. In addition to selected microdeletions, three studies simultaneously assessed the validity of screening tests for genome-wide copy number variations larger than 7 Mb using cfDNA, as CNVs are considered to be the cause of subchromosomal microabberations including microdeletions [21,22,23]. Twelve studies solely evaluated the genome-wide detection of CNVs. Most of them screened for previously undiagnosed fetal CNVs [4,31,32,33,34,35,36,37,38], but two studies obtained samples with known CNVs and retrospectively conducted NIPT analysis [39,40].

### 3.3. Patient Characteristics and Acquisition of Samples

Most of the studies obtained plasma samples from the population of pregnant women who underwent NIPT without specifically defining referral indications for the screening [4,10,15,17,19,20,21,28,30,31,33,34,35,37,38]. Some included only samples of women with high-risk pregnancies (over 35 years of age), positive serum screening results, a history of aneuploidy, abnormal ultrasound findings, or simply maternal anxiety [22,32,40]. Two studies analyzed samples that showed an increased risk of chromosomal abnormalities from an NIPT which had already been performed [16,18]. Four reports took into consideration samples with already-confirmed fetal microdeletions or CNVs by invasive testing and euploid samples as controls [12,14,29,39]. Only one study explicitly analyzed twin pregnancies [13]. The most common exclusion criteria in these studies were known parental chromosomal abnormalities, multiple pregnancies, known maternal malignancy, the mother receiving an allogeneic blood transfusion, organ transplantation surgery, stem cell therapy, or immunotherapy, as well as an egg donor or surrogate pregnancies [10,13,34,36,37,38]. Li et al. also excluded samples whose transportation to the laboratory took more than 48 h, those with visible hemolysis, and a fetal fraction of less than 3% [40]. Due to the low prevalence of chromosomal aberrations of interest, some researchers used in vitro created plasma samples constructed by using DNA from the affected individual, with known deletion or CNV, and spiking it into the isolated plasma DNA of non-pregnant women [11,14,24,25].

### 3.4. Molecular Methods for cfDNA Analysis

Nowadays, there are two primary next-generation sequencing-based approaches for cfDNA testing: massively parallel shotgun sequencing (MPSS), which sequences DNA fragments from the whole genome, and targeted sequencing, which is the selective testing of targeted genomic regions. Both techniques are based on counting sequenced DNA fragments obtained from maternal blood and consider only the number of reads to identify numeric abnormalities of fetal chromosomes. Another type of targeted cfDNA testing is single-nucleotide polymorphism (SNP) sequencing, which enables a more accurate cfDNA analysis, allowing for a qualitative approach to differentiating between maternal and fetal input. SNP sequencing can rely on the allele ratio or specifically target the amplification of polymorphic loci, followed by NGS and bioinformatics analysis. In such an approach, allelic information from both parents is included in the analysis, taking into consideration different genetic inheritance patterns [41].

A total of 21 studies used massively parallel sequencing technology that enables the detection of microdeletions and genome-wide CNVs without prior knowledge of the event’s location. After cfDNA quantification, libraries were tag-sequenced to generate 3.5 M–32 M reads per sample and aligned to the human reference genome [22,29]. Reads were then allocated to continuous non-overlapping 20 kb–100 kb bins and filtered to remove bins with abnormal GC content [13,21,33,38,39,40]. Next, similar statistical methods were used by five teams of researchers. They normalized the bin counts and then used the circular binary segmentation algorithm to divide each chromosome into contiguous regions of equal copy numbers. This step was followed by the identification of segments with consistently under-represented regions indicative of a loss in the genome. Furthermore, they used decision tree analysis to differentiate whole-chromosome events from deletions [15,22,23,25,28]. Two studies also described the combination of the MPSS method with technology that leverages the reduced size of fetal-derived cfDNA fragments, in comparison to maternally derived ones, to increase the sensitivity of the test. After quantification, they performed the size selection of cfDNA libraries by removing fragments > 200 nt via gel electrophoresis. These size-selected libraries contained a higher cffDNA fraction because, after the removal of maternally derived fragments, cffDNA represented a higher share of ultimately analyzed cfDNA. Importantly, the obtained gain in a fetal fraction was molecular and not algorithmic [20,31].

Targeted or directed technologies of cfDNA testing, in contrast to massively parallel shotgun sequencing, enable the detection of microdeletions and CNVs of known pathogenicity only, instead of testing the entire genome and consequently revealing CNVs of unknown significance [41].

In six included studies, samples were analyzed using an SNP-based screening methodology. For the detection of DiGeorge-causing microdeletions, sets of pooled primers containing 672 or 1351 SNPs were designed to target the 2.91 Mb section of the 22q11.2 region that constitutes approximately 87% of all deletions detected in individuals with 22q11.2 deletion syndrome [10,12,14,17]. Wapner et al. and Martin at al. also used sets of primers designed to amplify 1152 SNPs in each of the following regions: a 10 Mb region deleted in ~60% of patients diagnosed with 1p36 deletion syndrome, a 20 Mb region deleted in ~65% of patients diagnosed with Cri-du-chat syndrome, and a 5.85 Mb region deleted in ~28% of patients diagnosed with the Prader–Willi/Angelman syndromes [14,17]. Amplified samples were sequenced to 3.2–4.7 million reads per sample [12,17]. Deletions were predicted based on the allele distribution pattern for SNPs in the regions of interest [10,12,17]. Yang et al. used the target-captured SNP sequencing of cfDNA to detect pathogenic SEA deletion—the cause of α0-thalassemia. Nearly 2000 SNPs were used to target the gene region of alpha-globin (HBA) and 20,000 bp upstream and downstream of the gene region [26].

The droplet digital PCR-based method for the non-invasive detection of SEA deletion was described by Sawakawongpra et al. This technique amplifies a low initial amount of target DNA molecules and itemizes different PCR products using probe-specific fluorescent signals. Two probes were designed—one to bind the genomic region inside the targeted deletion and the other one to bind just outside of the SEA locus. The first signal was expected to be detected only in cases of a wild type of the gene, whereas the other one was expected to be present both in a wild type and in cases with a SEA deletion [27].

Schmid et al. developed a targeted microarray-based cfDNA test for the detection of 22q11.2 microdeletion. Additional 500 digital analysis of selected regions (DANSR) assays, in comparison to array-based NIPT for detecting common aneuploidies, were designed against targets uniformly distributed within a 3 Mb region of interest. Each of the samples was furtherly analyzed on a single custom microarray [11]. Despite its efficiency, microarrays are not commonly used in cfDNA testing.

Koumbaris et al. and Neofytou et al. developed novel analytical approaches using target capture sequences (TACS) to enrich regions of interest associated with sought-after microdeletions. Target loci were selected based on the GC content, the distance from repetitive elements, and the absence of known surrounding complex architecture. This type of approach avoids CNVs of unknown clinical significance and has the ability to identify deletions in a fetus as small as 0.5 Mb in size [19,24].

Two studies retrospectively analyzed NIPT results obtained by various molecular methods and performed solely confirmatory testing using invasively acquired samples. In many cases, while obtaining samples, Petersen et al. were not provided with the information of the laboratory that performed the NIPT. Consequently, they used pooled data for the interpretation of the results [16]. In the other study, Schwartz et al. broadly separated results depending on the NIPT technologies used. They noticed a statistically significant difference between a PPV of 4.2%, acquired by the usage of the SNP-based approach, and one of 32.3%, acquired by MPSS technology-based tests, but noticed that this was most likely false and caused by false-positive results due to the presence of the homozygotic stretches associated with consanguinity [18].

### 3.5. Study Outcomes

Studies that used MPSS technology to test for common microdeletions achieved an overall sensitivity of 85.4–97.2% and a specificity of 95.7–99.8% [20,28,29].

Regarding the individuality of positive predictive values (PPVs) for certain conditions, Helgeson et al. presented individual PPVs for microdeletions ranging from 100% for the Wolf–Hirschhorn, Jacobsen and Langer–Giedion syndromes, 96.9–100% for DiGeorge syndrome and 66.7% for Cri-du-chat syndrome. While testing for DiGeorge, 20/32 detected deletions had a maternal contribution. A likely explanation for this high rate of maternal findings lies in the small size of deletions detected in the 22q11 region which represents pregnant women with mild clinical findings [15]. Others, such as Liang et al., achieved a PPV ranging from 93% for DiGeorge to 0% for 1p36 microdeletion [21].

Clearly, one of the factors that affect the detection of subchromosomal deletions and CNVs using the whole-genome sequencing approach was the size of the event of interest. The larger the CNV in the cffDNA is, the easier it is to detect it against a background of normal maternal DNA [15]. In the study by Yin et al., 93.3% of deletions/duplications larger than 5 Mb and 100% larger than 10 Mb in size were detected at 3.5 million reads per sample. In contrast, only 1.2% of deletions/duplications less than 5 Mb in size and none less than 1 Mb in size were detected at 3.5 million reads per sample. Also, 67.2% of false positives predicted a deletion/duplication less than 5 Mb in size [29]. In the study conducted by Kucharik et al. out of the 1,705,600 carried out simulations, the simulated syndrome was correctly predicted in 937,335 cases, resulting in a sensitivity of 55.0%. Importantly, the sensitivity increased to 97.1% if the read count was at least 15 M and the size of the deletion was at least 3 Mb. The mutuality between the above-mentioned deletion size and the fetal fraction percentage was found to be a key parameter in NIPT, and different combinations of them were tested from 5% to 20%. Fetal fractions lower than 5% were shown to be problematic due to an increased number of false-negative detections. This approach achieved an accuracy of 79.3% for a 10% fetal fraction with a 20 M read depth, which further increased to 98.4% if the search was only for deletions longer than 3 Mb. The results of the in silico simulated data were in accordance with an artificial laboratory sample evaluation test that correctly detected 24 out of 29 control samples [25].

A majority of the included studies investigated the genome-wide detection of CNVs. When comparing these results, there is no consistency in achieved sensitivity, specificity, and PPV rates depending on the CNV size. While Lo et al. demonstrated a significantly higher sensitivity for the detection of CNVs larger than 6 Mb (83% compared to 20% for ones smaller than 6 Mb), whereas Chen et al. achieved the highest PPV for CNVs size of 5–10 Mb [34,39]. However, this was not the case in a study conducted by Yu et al. who for CNVs 5–10 Mb achieved the highest sensitivity (100% in comparison to 92% and 68% for CNVs > 10 Mb and <5 Mb) but the lowest PPV (71% in comparison to 85% and 81% for CNVs > 10 Mb and <5 Mb) [33]. One study demonstrated a significantly lower PPV, only 3%, for CNVs greater than 10 Mb, compared with 40% for the ones less than 10 Mb [4]. In general, smaller CNVs were more likely to be confirmed than larger CNVs. Furthermore, for the cases where two or more CNVs were identified, where only one of the findings was confirmed by diagnostic testing, the smaller one was confirmed more frequently than the larger finding [23]. 

In another study, deeper sequencing correctly identified the fetal CNV in 9 of 11 samples where the imbalance had not been detected by the standard shallow-sequencing pipeline. The discrepancy in the count ratios decreased as the depth of sequencing increased, as demonstrated by one fetus with a 22q11.2 deletion, which was ultimately detected when the sample was sequenced to a depth of 32 million reads. In addition to identifying the CNVs, the pipeline indicated locations that were highly accurate and matched well with positions provided by microarray analysis [39]. The study by Rafalko et al. demonstrated the importance of the initial ultrasound fetal risk assessment, as cases referred to due to ultrasound findings as the sole indication for testing comprised 15% of the overall screening cohort, compared to 33% for the CNV-positive cohort. For cases in which only one of the two findings was confirmed, the detected CNV that was discordant showed sequencing data suggestive of mosaicism. This may have been caused by a segmental “rescue” event in progress, such as telomere capture, which acts to stabilize an open deletion by acquiring material from another chromosome and results in mosaicism for the “stabilizing” CNV [23].

A study which used the SNP-based approach scored detection rates of 97.8% for a 22q11.2 deletion and 100% for the Prader–Willi, Angelman, 1p36 deletion, and Cri-du-chat syndromes. The false-positive rates were 0.76% for 22q11.2 deletion syndrome and 0.24% for Ci-du-chat syndrome. No false positives occurred for the Prader–Willi, Angelman, or 1p36 deletion syndromes. An explanation for the lower DR for the 22q11.2 locus lies in the fact that the number of SNPs targeted in this region was less than for other locations. The performance of this SNP-based method for the detection of well-defined microdeletions is expected to depend primarily on the number of informative SNPs in each region of interest, which may limit its detection capabilities for small regions of interest [14]. The study by Martin et al. first achieved a positive predictive rate of 15.7% for 22q11.2 deletion syndrome, and 5.2% for the other four disorders combined. Then, the analysis of high-risk samples with a revised protocol of high-depth sequencing showed an increase in the PPV rate to 44.2% for 22q11.2 and 31.7% for the others as well as a decrease in false-positive rates [17]. In the studies analyzing only deletions characteristic of DiGeorge syndrome, a sensitivity and specificity of 90% and 99.74%, respectively, were presented [12]. Overall, the PPV was 18%. In contrast, for cases with no abnormal ultrasound findings prior to NIPT, the PPV was 4.9%. This estimate was based on a small sample size and could be subject to an ascertainment bias because information about ultrasound findings was not comprehensively gathered at the time of testing [10].

The first study to show the application of ddPCR to identify the copy number of SEA deletion in unprocessed cell-free DNA obtained from maternal plasma yielded a sensitivity of 95.38% and a specificity of 91.01% [27].

A prenatal screening test for 22q11.2 deletion using a targeted microarray-based cfDNA test achieved a sensitivity of 75.4% and a specificity of at least 99.5%. The smallest deletion size detected was 1.96 Mb. There was no interdependence between the sensitivity and deletion size. To comprehend these results, it is crucial to take into consideration the fact that this study included samples with the typical 3 Mb deletion as well as samples with smaller nested deletions, unlike the other included studies. In fact, one could argue that the sensitivity reported for comparable cfDNA tests using MPSS and SNP technologies should be adjusted if established only based on the common 3 Mb 22q11.2 deletion [11]. Both of the studies which used NIPT based on a validated targeted capture enrichment technology identified all microdeletions correctly without any false-negative events, exhibiting 100% sensitivity and 100% specificity. This type of approach avoids CNVs of unknown clinical significance and has the ability to identify deletions or duplications in a fetus as small as 0.5 Mb in size [19,24].

Studies that used retrospectively obtained data from laboratories using various NIPT platforms yielded an overall PPV of 9.2–13% [16,18]. In the study conducted by Schwartz et al., 39.3% of the cases in which microdeletion was confirmed by invasive testing had additional abnormal microarray findings. Unrelated abnormal microarray findings were detected in 11.8% of the patients in whom the screen positive microdeletion was denied and declared as false positive [18].

### 3.6. Limitations and Biases

The limitations of our systematic review largely reflect the shortcomings of the reports reviewed. The weaknesses of included studies arose from the their processes for gathering each cohort, choices of technology for the analysis of collected or artificially created samples, follow-up processes, as well as the biological characteristics of the subchromosomal aberrations of interest.

Firstly, diverse indications for wide NIPT performance were applied depending on the countries the studies were conducted in, and this may have influence the calculated PPVs and sensitivities. The sample size in a substantial number of the studies was too small for comprehensive analysis. In addition, the gestational age of a large number of the recruited pregnancies was in the second trimester, resulting in a higher fetal fraction than in cases of samples obtained in clinical practice [12,14,40]. As the incidence of analyzed subchromosomal aberrations is extremely low, to decrease the cost of the research, several studies included only high-risk populations and this way may have introduced bias while calculating the PPV [23]. Due to the unknown fetal prevalence of screened microdeletions and CNVs, it was difficult to estimate the PPV of the molecular methods used for the analysis [39]. In some studies, microduplication screening results were calculated in summarized PPV and sensitivity values [24,29]. The studies that evaluated prenatal screenings for CNVs where similar in this regard, since they are not only the cause of microdeletions but also other submicroscopic chromosomal rearrangements. Retrospective cohort studies often lack sufficient data such as the total number of cfDNA tests performed during a given period, meaning they lack the total denominator required for the evaluation of the test’s specificity. Also, two such studies did not take into consideration differences between the testing platforms used for the analysis of samples when calculating overall statistics [16,18]. Furthermore, some of the researchers did not have the access to the clinical data to assess possible explanations for false-positive results such as the presence of a vanished twin’s cfDNA in the maternal plasma, placental mosaicism, maternal chromosomal abnormalities, or maternal neoplastic conditions [16,18,22,23,36]. Studies that used artificial samples did not take into consideration real case scenario causes of inconsistent results when expressing sensitivity, specificity, and PPV [11,24,25]. Most of the included studies could correctly address the test sensitivity, as they were limited by the incomplete follow-up of the pregnancy outcome of negative cfDNA screening cases and ascertainment bias [10,11,16]. The inconsistent results may be related to the GC bias [30]. NIPT methodology in general suffers from the problem of multiple hypothesis testing, meaning that false-positive rates become additive for independently analyzed genome regions [22]. Several screen-positive cases were validated by low-pass whole-genome sequencing and fluorescence in situ hybridization rather than chromosomal microarray analysis—the gold standard diagnostic for microdeletions [10,12,33].

The extensive and systematic literature search, which included a backward citation search that yielded additional reports, is one of the strengths of this study. An assessment of the methodological quality of the included studies was not performed after taking into consideration the number of included studies and the extent of the information that would have arisen from this, which would have exceeded the scale of this systematic review. The possibility of pooling data for meta-analysis was explored but this was not pursued because of the heterogeneity of the studies with respect to the processes for gathering the cohorts, molecular methodologies used, and confirmation testing performed. Therefore, the conclusions were based on narrative synthesis. Between-study heterogeneity was not assessed quantitatively, as a meta-analysis could not be conducted.

## 4. Discussion

CNVs are the cause of microdeletions—structural chromosomal abnormalities whose size is less than 5 Mb—a standard resolution of karyotyping and therefore cannot be detected by this method [2]. Although individually extremely rare, with frequencies ranging from 1:4000 for DiGeorge syndrome to 1:50,000 for Cri-du-chat syndrome, the overall prevalence rate of microdeletions is considered to be around 1:2500 and is not associated with maternal age [7,42]. Hence, for the population of pregnant women younger than 35 years, fetal microdeletions are more common than Down syndrome and often show equally serious phenotypes [43]. This imposed the need for a form of preinvasive testing for these conditions. With CMA being the gold standard diagnostic method but exposing pregnant women to possible complications due to the invasive procedures involved in the obtaining of samples, NIPT technology slowly but surely became a leading method in prenatal screening for microdeletions and CNVs [40].

Cell-free DNA-based screening tests have been developed and marketed exclusively by the developers to the practitioner and patient communities without conducting independent trials and validating their performance prior to their introduction into the marketplace [43]. The process of developing a screening test from the laboratory to clinical application requires the determination of its validity and utility. In the context of NIPT, the assessment of analytical validity answers the question of whether various concentrations of placental and maternal DNA can be used to determine the presence or absence of a condition of interest. Clinical validity refers to the detection rate, sensitivity, and specificity of the evaluated test—metrics that are independent of the prevalence of the condition screened for. Clinical utility evaluates the practical usefulness of the test to the screened population, expressed through objective metrics such as the PPV and NPV. The American College of Medical Genetics and Genomics, in their position statement from 2016, claims that the previously mentioned values should be expressed for each CNV screened when reporting laboratory results [44]. Unfortunately, it is fair to say that the selection of microdeletions and CNVs included in commercial panels was often more driven by their detection feasibility than by their clinical relevance [2].

As previously mentioned in Section 3.4, there are two primary approaches for cfDNA-based screening tests. Targeted sequencing technologies that process a smaller amount of data provide better values in terms of the test’s validity and utility indicators, requiring less time and expense than MPSS-based methods [7,28]. Increasing the sequencing depth is the most direct way to improve the accuracy of diagnosis. In clinical practice, this is limited by the sequencing cost. However, certain parts of the genome may not be amenable to adequate coverage because of the inherent features of DNA structure (for example GC rich regions and heterochromatin regions of repetitive DNA sequences) [29]. Kucharik et al. recommend using approximately 16 M–17 M reads per sample for analyses, due to fact that the detection rate reaches a plateau for a 10% fetal fraction and 3 Mb deletion size around this point [25].

Other than the sequencing depth, the detection capability is dependent on the variation or microdeletion size, fetal fraction, as well as the biological variability of the region of interest [7,28]. Reports claim that the detection efficiency for microdeletions and CNVs is determined mainly by their size, but in the studies by Yu et al. and Chen et al., the highest PPV and sensitivity were achieved for CNVs between 5 and 10 Mb in size in comparison to smaller and larger ones [29,33,34,39]. This may be explained by the limited number of cases identified in that subgroup and the case bias that possibly contributed to a higher sensitivity [33,34].

Although there were initially concerns that the technical aspects of the sequencing methods and bioinformatics analyses were the reason for the reduction in specificity and false-positive results, over time, it has become clear that a significant proportion of cases have an underlying biological etiology [45]. CffDNA is of placental origin, and, consequently, a confined placental mosaicism observed in around 1% of all pregnancies can be the cause of FP results. Other potential causes are maternal microdeletions and CNVs; Lo et al. described the detection of all three maternal microduplications but could not determine if the fetus had inherited them. Therefore, achieving sufficient accuracy in fetal inheritance would require knowledge of fetal fractions and the use of counting statistics [39]. Also, in the case of multiple reported abnormalities, the source of pathologically altered DNA could be apoptotic tumor cells of maternal origin [46]. Rafalko et al. reported that at least 12 of their positive cases were caused by maternal fibroids and myelodysplastic syndromes [23]. Another possible source of pathological findings is the presence of a vanished co-twin’s DNA, although this extremely rare [45].

Despite the detection rates of all the cfDNA-based screening tests being high, the PPV depends on the patient’s *a priori* risk for the analyzed disorder, which is primarily determined by the prevalence of an abnormality [1]. With the possible exception of 22q11.2 and 1p36 deletions, microdeletions show no phenotypic characteristics detectable by ultrasound, and screening is essentially performed on an average-risk population of pregnant women. As the adequate assessment of clinical utility is difficult while using the traditional idea of prospective randomized trials due to the rarity of the microdeletions and CNVs, study designs have used archived samples and artificial mixtures of abnormal DNA to provide at least an estimate of accuracy [11,24,25,47]. The current systematic review was unable to provide any new contributions to this topic considering that the analyzed reports included fetuses with different background risks, and, consequently, the reported PPVs may not reflect the genuine clinical utility.

None of the included studies performed systematic confirmatory analysis by CMA for negative/low-risk cases. They mostly relied on clinical follow-up. That being the case, the exact negative predictive values could not be determined. In an average-risk population, an estimated NPV of 99% is more often than not the result of the rarity of the condition rather than the test performance itself. Yaron et al. made a calculation that, given a 1.7% *a priori* risk of any clinically significant microdeletion or CNV being present in the fetus, a negative NIPT result would only modestly reduce the risk to 1.6% [47].

One of the limitations of implementing NIPT methods in clinical practice is the fact that the fetal genome is screened for specific chromosomal abnormality and not a consequently expressed clinical syndrome. For example, DiGeorge syndrome is not caused by a single chromosomal entity but rather a group of different microdeletions, all located in the 22q11.1 chromosome band. Approximately 85% of the cases are caused by a typical 3 Mb size deletion that encompasses 45 functional genes located between the low copy repeats LCR22A and LCR22D, which, respectively, correspond to the SNP coordinates 18,835,221 and 21,592,477 (based on human reference genome hg19) [10,11]. The remaining patients suffering from DiGeorge syndrome have atypical or nested microdeletions that occur between other low-copy repeats within the same region [11,48]. Even though, in the study by Schmid et al., atypical and nested deletions were covered by DANSR assays, neither of them is detectable by currently available commercial tests [11,12]. Similarly, only 65–75% of Prader–Willi cases are caused by microdeletion, whereas the remaining cases are caused by uniparental disomy or single gene disorder [8]. In addition, the variable penetrance of a considerable number of microdeletions may lead to milder phenotypic expressions of the same genetic defect [9].

The debate about the optimal way to implement wide NIPT into clinical practice to ameliorate the management of pregnancy is still ongoing. The main obstacle remains the undefined reliability of these tests. The current position of the main professional organizations, namely, the American College of Obstetricians and Gynecologists, the American Society for Human Genetics, and the European Society for Human Genetics, is that NIPT is not recommended for the detection of microdeletions. Alternatively, the American College of Medical Genetics and Genomics holds an opinion that informing women about the availability of cfDNA-based screening for selected microdeletions should be provided when specific conditions are met by both the healthcare provider and the performing laboratory [1,49]. This configures NIPT for microdeletions and CNVs as contingent tests offered in cases of pathological ultrasound findings or abnormal serum marker levels along with anamnestic indications for screening.

It is necessary to provide comprehensive genetic counseling to all pregnant women undergoing NIPT. Special attention should be paid to CNVs classified as variants of unknown significance as well as all the other limitations of the test arising from the biological characteristics of analyzed genetic abnormalities and the molecular methods used for the analysis. The main limitations to the introduction of these tests into clinical practice are the associated cost, which still exceeds those of other prenatal screening methods, and a high share of false results, leading to challenges in the management of these cases [3].

## 5. Conclusions

Considering the limited follow-up and validation data available at this time, NIPT for microdeletions and CNVs should be used with caution and screen-positive results confirmed by invasive testing. Any developments regarding new technologies should undergo robust evaluation in terms of validity and clinical utility. The commercial implementation of NIPT should be subordinate to the public health sector. Standards for the inclusion of cfDNA-based screening methods into national health systems should be established by major organizations in the field of prenatal diagnostics.

## Figures and Tables

**Table 1:** Studies included in the systematic review.

Study	Country	Type of Study	Microdeletions/CNVs	Molecular Method	Number of Participants	Sample	Size	Number of Reads	TP	FP	PPV	Sensitivity	Specificity
Yang et al., 2019 [26]	China	training set-retrospective, testing set-prospective cohort	SEA deletion	targeted method (SNP-based)	878	plasma	20 kb	training set 4.84 M, testing set 5.22 M	321	16	95.25%	98.98%	96.06%
Sawakwongpra et al., 2021 [27]	Thailand	prospective cohort	SEA deletion	targeted method (droplet digital PCR)	22	plasma	20 kb					95.38%	91.01%
Gross et al., 2016 [10]	USA	retrospective cohort	DiGeorge	targeted method (SNP-based)	21,948	plasma	2.91 Mb	8.9 M	11	50	18%		
Schmid et al., 2018 [11]	UK	cross-sectional	DiGeorge	targeted method (microarray-based)	1953	plasma + artificial	1.96–3.25 Mb		97	8	92.38%	75.2%	99.6%
Ravi et al., 2018 [12]	USA	prospective cohort	DiGeorge	targeted method (SNP-based)	10 affected and 409 controls	plasma	2.55–3.16 Mb	4.7 M			19.6%	90%	99.74%
Lin et al., 2021 [13]	Taiwan	retrospective cohort	DiGeorge	MPSS	8158	plasma	3 Mb	20 M	7	6	53.85%	100%	99.92%
Wapner et al., 2015 [14]	USA	prospective cohort	microdeletions (common)	targeted method (SNP-based)	6 affected, 352 controls, 111 artificial	plasma + artificial	2.91–20 Mb	8.9 M	106	4	96.36%		
Zhao et al., 2015 [28]	USA	prospective cohort	microdeletions (genome-wide)	MPSS	178	plasma	3–40 Mb	0.2× coverage	17	1	94.4%	94.4%	99.4%
Helgeson et al., 2015 [15]	USA	prospective cohort	microdeletions (common)	MPSS	175,393	plasma					90.9%		
Yin et al., 2015 [29]	China	prospective cohort	microdeletions and microduplications (genome-wide)	MPSS	1476	plasma	0.52–84 Mb	3.5 M	56	58	49.12%	85.4%	95.7%
Petersen et al., 2017 [16]	USA	retrospective cohort	microdeletions (common)	various technologies	712	plasma	>1.5 Mb		7	45	13.4%		
Martin et al., 2018 [17]	USA	retrospective cohort	microdeletions (common)	targeted method (SNP-based)	114,616	plasma	2.91–20 Mb	>3.2 M	30	43	41.1%	96.77%	81.62%
Schwartz et al., 2018 [18]	USA	retrospective cross-sectional	microdeletions (common)	various technologies	349	plasma			25	310	7.4%		
Hu et al., 2019 [30]	China	prospective cohort	microdeletions (genome-wide)	MPSS	8141	plasma	>10, <10 Mb	4.89 M	13	23	36.11%		
Koumbaris et al., 2019 [19]	Cyprus	retrospective cohort	microdeletions (common)	targeted method (TACS)	2033	plasma			5	0	100%	100%	100%
Welker et al., 2021 [20]	USA	prospective cohort	microdeletions (common)	MPSS (FFA method)	2401	plasma						97.2%	99.8%
Pescia et al., 2017 [31]	Switzerland	retrospective cross-sectional	CNVs	MPSS	6388	plasma		>10 M	7	3	70%		
Lo et al., 2016 [39]	UK	prospective cohort	CNVs	MPSS	31 affected + 534 controls	plasma	>6, <6 Mb	4–10 M			55%	83%	99.6%
Li et al., 2016 [40]	China	prospective cohort	CNVs	MPSS	117	plasma	>5, <5 Mb	3.95 M	11	4	73.33%	61.1%	95%
Lefkowitz et al., 2016 [22]	USA	retrospective cross-sectional	CNVs > 7 Mb and common microdeletions	MPSS	1166	plasma	>7 Mb + selected smaller	32 M	42	1	97.67%	97.7%	99.9%
Fiorentino et al., 2017 [32]	Italy	prospective cohort	CNVs	MPSS	12,114	plasma	>1.9 Mb	30 M	8	5	61.54%	100%	99.96%
Yu et al., 2018 [33]	China	prospective cohort	CNVs	MPSS	20,003	plasma	>10, 5–10, <5 Mb	4.2 M	29	7	80.56%	80.56%	
Liang et al., 2019 [21]	China	prospective cohort	CNVs and common microdeletions	MPSS	94,085	plasma	>10, <10 Mb	20 M	49	71	40.8%	90.74%	99.92%
Chen et al., 2019 [34]	China	prospective cohort	CNVs	MPSS	42,910	plasma	>10, 5–10, <5 Mb		20	49	28.99%		
Luo et al., 2020 [36]	China	retrospective cohort	CNVs	MPSS	40,256	plasma		>3.5 M	4	131	3%		
Pei et al., 2020 [4]	China	retrospective cohort	CNVs	MPSS	141	plasma	>20, 10–20, <10 Mb	>6 M	21	120	14.89%		
Liu et al., 2020 [37]	China	retrospective cohort	CNVs	MPSS	42,924	plasma			11	27	28.95%		
Rafalko et al., 2021 [23]	USA	prospective cohort	CNVs > 7 Mb and common microdeletions	MPSS	86,902	plasma	>7 Mb + selected smaller		181	63	74.2%		
Chen et al., 2021 [35]	China	prospective cohort	CNVs	MPSS	34,620	plasma	>5 Mb	0.1× coverage	21	20	51.22%		
Lai et al., 2021 [38]	China	prospective cohort	CNVs	MPSS	86,262	plasma	6–32.5 Mb	3 M	4	8	33.3%	20%	99.99%
Neofytou et al., 2017 [24]	Cyprus	prospective cohort	common microdeletions + Potocki Lupski	targeted method (TACS)	21 affected + 50 controls	plasma + artificial	>0.5 Mb		21	0	100%	100%	100%
Kucharik et al., 2020 [25]	Slovakia	case-control study	microdeletions (common)	MPSS	29	artificial	0.9–21 Mb	20 M	24	0	100%		

CNV—copy number variation, PPV—positive predictive value, TP—true positive, FP—false positive, SEA—Southeast Asian, SNP—single nucleotide polymorphism, PCR—polymerase chain reaction, MPSS—massively parallel shotgun sequencing, TACS—target capture sequences, FFA—fetal fraction amplification, USA—United States of America, UK—United Kingdom.

## Data Availability

The data that support the findings of this study are available upon request from the corresponding author.
